# Clinicopathological significance of WIF1 hypermethylation in NSCLC, a meta-analysis and literature review

**DOI:** 10.18632/oncotarget.13707

**Published:** 2016-11-29

**Authors:** Hao Guo, Shuni Zhou, Lili Tan, Xiaoyu Wu, Zhenfeng Wu, Ruizhi Ran

**Affiliations:** ^1^ Department of Oncology, The Central Hospital of Enshi Tujia and Miao Autonomous Prefacture, Enshi, Hubei 445000, China; ^2^ Department of Chinese Medicine and Cardiology, The Central Hospital of Enshi Tujia and Miao Autonomous Prefacture, Enshi, Hubei 445000, China; ^3^ Surgical Oncology, Affiliated Hospital of Nanjing University of Traditional Chinese Medicine, 1 Nanjing 210029, China

**Keywords:** NSCLC, WIF-1, methylation, tumor suppressor gene, drug target

## Abstract

Methylation of the *WIF-1* gene can lead to the loss of *WIF-1* expression which has been observed in numerous types of cancer including NSCLC. However, the association and clinicopathological significance between *WIF-1* promoter hypermethylation and NSCLC remains unclear. In the present study, we performed a meta-analysis to evaluate the clinicopathological significance of *WIF-1* hypermethylation in NSCLC. A systematic literature search was carried out using Pubmed, EMBASE, Web of Science and CNKI. The Cochrane software Review manager 5.2 was used. The frequency of *WIF-1* hypermethylation was significantly increased in NSCLC compared with normal lung tissue; the pooled OR was 8.67 with 95% CI 1.64-45.88, *p* = 0.01. The rate of *WIF-1* hypermethylation was higher in SCC than in AC, OR was 1.74 with 95% CI 0.97-3.11, *p* = 0.06. In addition, *WIF-1* loss was correlated with low 5-year survival rate. In summary, *WIF-1* *hyper*methylation is a potential biomarker for diagnosis of NSCLC. *WIF-1* hypermethylation is predominant in squamous cell carcinoma (SCC), suggesting that *WIF-1* methylation contributes to the development of NSCLC, especially SCC.

## INTRODUCTION

Lung cancer has been the leading cause of cancer-related mortality worldwide. [1] Lung cancer can be classified two major histological groups, small cell cancer and non-small cell lung cancer (NSCLC). NSCLC can be divided into adenocarcinoma (AC), squamous cell carcinoma (SCC), large cell carcinoma and others. [2] The overall 5-year survival rate of NSCLC remains less than 18%, [3] because a high proportion of NSCLCs are diagnosed at advanced stages. Therefore, it is particularly important to identify molecular markers for early diagnosis and determining prognosis.

Aberrant activation of the Wingless-type protein (Wnt) signaling pathways plays a very important role in the development of a variety of human cancers such as head and neck carcinoma, [4] melanoma, [5] colorectal cancer, [6, 7] and leukemia. [8] Previous evidence indicates that the inhibition of Wnt-1 induces apoptosis and suppresses tumor growth in lung cancer cell lines. [9–12] Wnt antagonists includes the secreted frizzled-related protein (sFRP) family, Wnt inhibitory factor-1 (WIF-1), and Cerberus, [13] Disabled 2 (Dab2) [14] and Dickkof (DKK) family [15]. WIF-1 has been identified as an important Wnt antagonist which inhibits Wnt/β-catenin signaling by directly binding to Wnt proteins. Methylation of the *WIF-1* gene can lead to the loss of WIF-1 expression which has been observed in numerous types of cancer including NSCLC. [16–20] However, the association and clinicopathological significance between *WIF-1* promoter hypermethylation and NSCLC remains unclear. In this study, we aim to systematically investigate the clinicopathological significance of *WIF-1* promoter hypermethylation and NSCLC and quantify the association between *WIF-1* promoter hypermethylation and NSCLC using meta-analysis methods. In addition, we summarize these findings and discuss the tumor suppressor function, as well as the clinicopathological significance of WIF-1 in NSCLC.

## RESULTS

**Figure 1:**
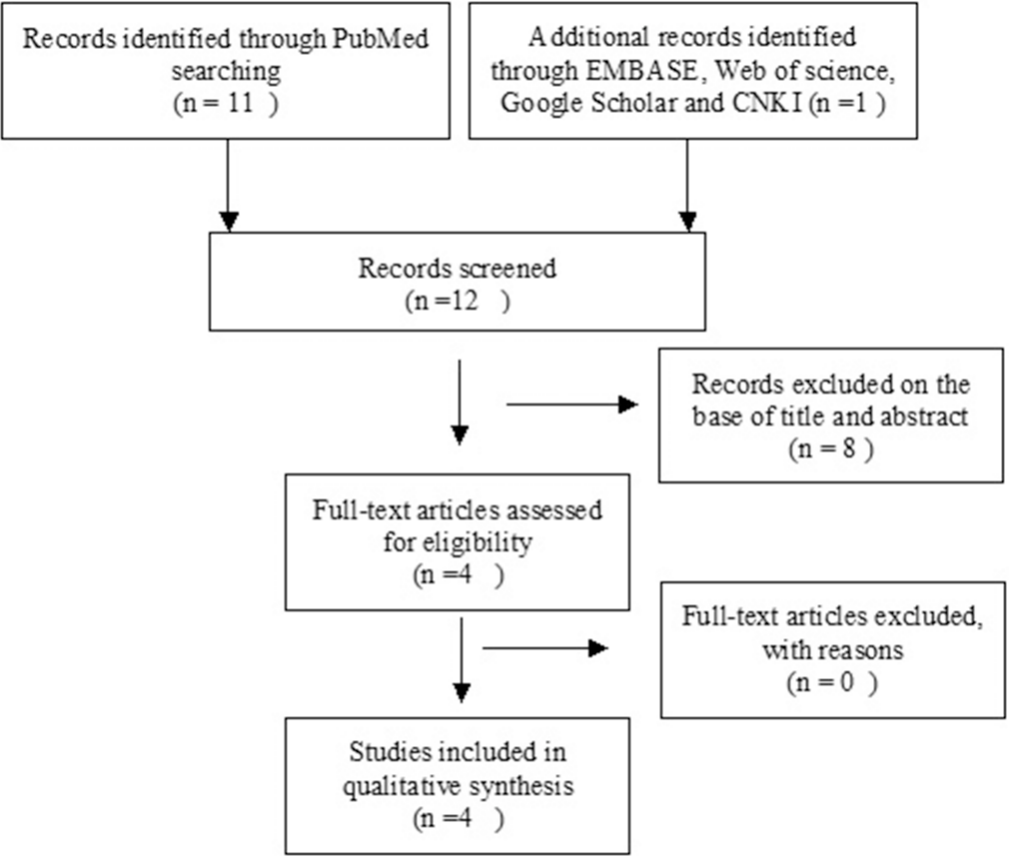
Schematic flow diagram for selection of included studies

Flow chart for study selection is reported in Figure 1. There were four relevant articles available for meta-analysis, which included 392 patients (Table 1, Table 2).

**Table 1:** All 12 studies identified through database searching

Author	Included/Excluded	Comments
Xie et al. 2015 [33]	Excluded	Norcantharidin inhibits Wnt signal pathway via promoter demethylation of WIF-1 in human NSCLC.
Xu et al. 2015 [34]	Excluded	Bisdemethoxycurcumin effects on TGF-β1 induced EMT in NSCLC are mediated through WIF-1
Tan et al. 2013 [32]	Excluded	miR-29s suppress the Wnt signaling pathway through demethylation of WIF-1 in NSCLC.
Liu et al. 2011 [35]	Excluded	Hypomethylation effects of curcuminoid on *WIF-1* promoter in NSCLC cell lines.
Liu et al. 2011a [12]	Excluded	Hypomethylation agent induces apoptosis in human NSCLC
Suzuki et al. 2010 [36]	Included	Molecular Characterization of Chronic Obstructive Pulmonary Disease-Related NSCLC through *WIF-1*aberrant methylation and Alterations of EGFR Signaling.
Gao et al. 2009 [37]	Excluded	The role of Epigallocatechin-3- gallate in the reversal of *WIF-1* promoter methylation in NSCLC cell line
Gao et al. 2009a [38]	Excluded	Procaine and procainamide inhibit the Wnt canonical pathway by promoter demethylation of *WIF-1* in lung cancer cells
Yoshino et al. 2009 [20]	Included	Promoter hypermethylation of the *p16* and *WIF-1*genes as an independent prognostic marker in stage IA NSCLC.
Yang et al. 2009 [39]	Excluded	The role *WIF-1* promoter hypermethylation in the diagnosis of NSCLC-related malignant pleural effusion.
Ren et al. 2007 [9]	Included	The relationship between WIF-1, Gsk-3β and nm23-H1 expression and the prognosis in patient with non-small cell lung cancer.
Mazieres et al. 2004 [19]	Included	Promoter Hypermethylation in Human lung cancer.

**Table 2:** Main characteristics of included studies

Author	Country	Sample (M/T)		Methylation in NSCLC(%) Avg. 26.78%	*WIF1* Methylation (M/T)		*WIF1* Methylation (M/T)		Wif1 Expression in NSCLC	Methylation site	Methods
		NSCLC	Normal		Smoking	Non-smoking	Male	Female			
Suzuki	Japan	42/177	4/80	23.7	48/162	14/67	46/160	16/69	N/A	Promoter	MSP
Yoshino	Japan	7/44	2/32	15.9	5/22	2/10	4/22	3/22	N/A	Promoter	MSP
Ren	China	N/A	N/A		N/A		N/A		N/A	Promoter	MSP
Mazieres	USA	15/18	0/18	83.3	N/A		N/A		3/18	Promoter	MSP

Abbreviations: NSCLC: Non-small cell lung cancer, M: Methylation, T: Total, N/A: Not available, MSP: Methylation-specific PCR

The quality of each study was assessed with the Newcastle Ottawa Quality Assessment Scale (NOQAS). Of the studies, two scored eight points, one scored seven points and one scored six points. Hence, the studies were of a relatively high quality (data not shown).

**Figure 2:**
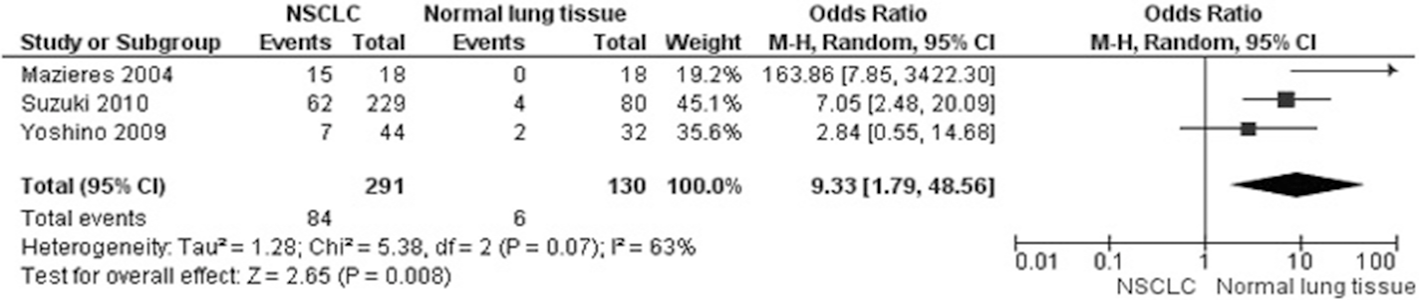
Forest plot for WIF-1 hypermethylation in NSCLC and non-neoplastic lung tissue The squares represent the weight of individual study in the meta-analysis, the line width indicates the corresponding 95% CI, The diamond represents the pooled OR, and the width of diamond indicates 95% CI.

**Figure 3:**
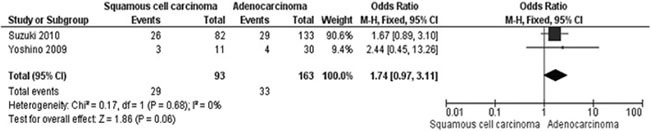
Forest plot for WIF-1 hypermethylation in AC and SCC The squares represent the weight of individual study in the meta-analysis, the line width indicates the corresponding 95% CI, The diamond represents the pooled OR, and the width of diamond indicates 95% CI.

**Figure 4:**
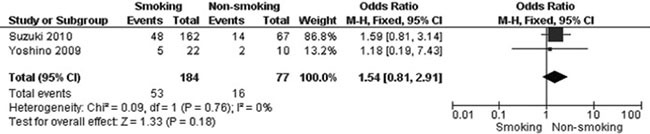
Forest plot for WIF-1 hypermethylation of NSCLC in smoking and non-smoking individual The squares represent the weight of individual study in the meta-analysis, the line width indicates the corresponding 95% CI, The diamond represents the pooled OR, and the width of diamond indicates 95% CI.

The frequency of *WIF-1* hypermethylation was significantly higher in NSCLC than in normal lung tissue, the pooled OR was 8.67 with 95% CI 1.64–45.88, z = 2.54, *p* = 0.01 (Figure 2). The rate of *WIF-1* hypermethylation was increased in SCC compared with AC, closely approaching the statistical significance, OR was 1.74 with 95% CI 0.97–3.11, z = 1.86, *p* = 0.06 (Figure 3). The rate of WIF-1 hypermethylation was not significantly associated with smoking behavior, OR was 1.54 with 95% CI 0.81–2.91, z = 1.33, *p* = 0.18 (Figure 4).

**Figure 5:**
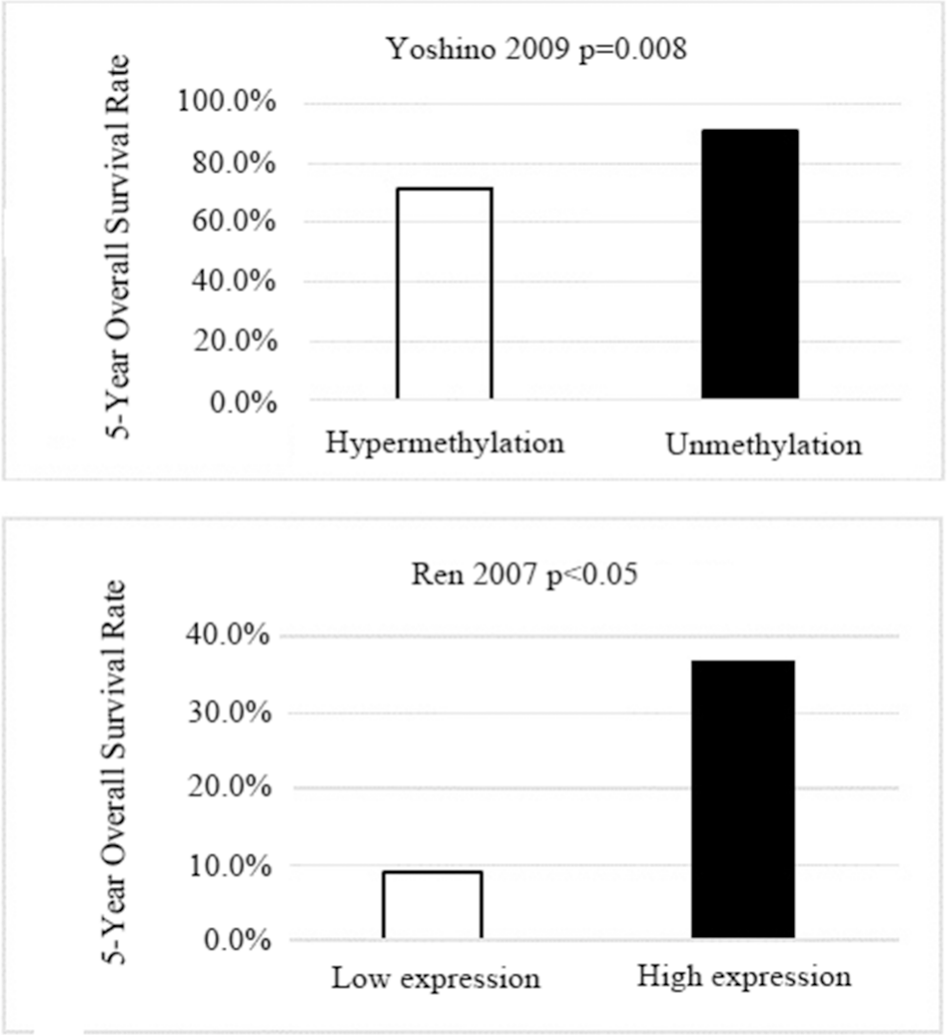
Charts for the association between WIF-1 expression or WIF-1 hypermethylation and 5-year survival rate

Two studies [9, 20] investigated the correlation between WIF-1 expression or methylation status and 5-year overall survival, indicating that NSCLC patients with low WIF-1 expression or *WIF-1* positive methylation were found to have a significantly lower rate of 5-year overall survival compared to the patients with high WIF-1 expression or *WIF-1* negative methylation in their tumor tissues (Figure 5).

**Figure 6:**
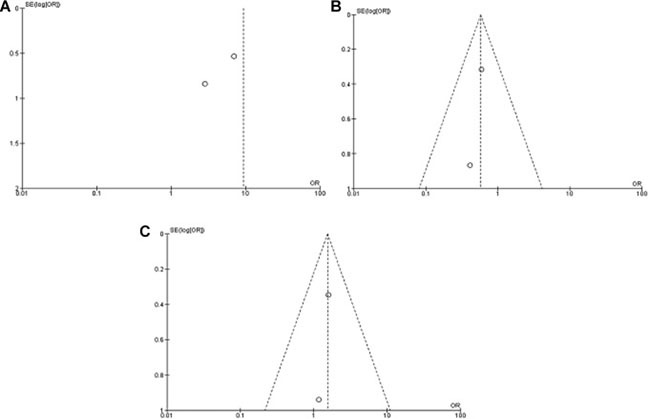
Funnel plot for publication bias (**A**) *WIF-1* hypermethylation in NSCLC and non-neoplastic lung tissue; (**B**) *WIF-1* hypermethylation in AC and SCC; (**C**) *WIF-1* hypermethylation of NSCLC in smoking and non-smoking individual. S.E., standard error; Area of the circle represents the weight of individual study.

A sensitivity analysis was conducted by removing one study from the meta-analysis at a time; the overall results were not significantly affected. The pooled ORs were not significantly changed, indicating the stability of our analyses. The funnel plots were largely symmetrical (Figure 6A, 6B, 6C), suggesting there were no publication biases in the meta-analysis of *WIF-1* methylation status between NSCLC and normal lung tissue, as well as *WIF-1* methylation status between AC and SCC respectively.

## DISCUSSION

Wnt signaling has been shown to control diverse biological processes and functions, including embryonic development, tissue regeneration, hematopoiesis, survival, cellular proliferation, and differentiation. [21–23] There are several Wnt inhibitors, including sFRPs, [24] WIF-1, [25] Cerberus [26] and Dab2 that inhibit Wnt signaling by competing with the binding of Wnt proteins to the Fz receptor. [13, 27] Another group of Wnt inhibitors are the members of the Dkk family that inhibit Wnt signaling by binding to the LRP5/LRP6 component of the Wnt receptor complex. [13] Downregulation of WIF-1 has been demonstrated in several type cancers including NSCLC and has been observed by immunochemistry in 60% of breast cancers and 75% of lung cancers. [28] A few studies showed that *WIF-1* silencing correlates with hypermethylation of its promoter in both cell lines and human NSCLC primary tissue; however, the rate of *WIF-1* promoter hypermethylation in NSCLC was diverse. For the first time we conducted a meta-analysis to more precisely evaluate the rate of *WIF-1* promoter hypermethylation in NSCLC and normal lung tissue. Our findings indicate the frequency of *WIF-1* promoter hypermethylation was 8.67 times higher in NSCLC than in normal lung tissue. Therefore, *WIF-1* promoter methylation could serve as a molecular diagnostic biomarker for NSCLC. On the other hand, since these are methylation marker, demethylation agents such as 5-Aza-2-deoxycitidine (5-AZA), an inhibitor of DNA methyltransferase can recover such epigenetic changes. [29,30] In addition, miR-29 family members (which downregulate the DNA methyltransferases DNMT3A and DNMT3B), as well as demethylation agents decreased promoter methylation and increased expression of WIF-1, as a result, those agents suppressed tumor proliferation and induced apoptosis in lung cancer cell lines. [11,12,31] Therefore, WIF-1 could be a novel drug target for the development of personalized therapy. Although further study is needed, our findings may contribute to the improvement of treatment and prognosis in patients with NSCLC.

In addition, our result showed that the frequency of *WIF-1* hypermethylation was increased in SCC than in AC, approaching more closely significance (*p* = 0.06). The prevalence of *WIF-1* hypermethylation in SCC may related to smoking behavior, as smoking is considered a risk factor for the development of SCC. However, *WIF-1* hypermethylation was not significantly higher in smoking NSCLC patients. Our analysis was limited by the small number of available studies. A large number of NSCLC patients are needed to more precisely evaluate the correlation between *WIF-1* hypermethylation and smoking in the future.

Two studies [9,20] showed that the loss of WIF-1 was correlated with the prognosis in patients with NSCLC; both studies revealed that the loss of WIF-1 was strongly associated with shorter 5-year survival. Yoshino et al. performed a multivariate analysis showing that *WIF-1* hypermethylation was an independent prognostic factor in relapse-free survival after adjusting age, gender, tumor size and histology. Therefore, loss of WIF-1 may increase the recurrence potential and malignant feature of NSCLC. Suzuki et al. reported the hypermethylation of *Wif1*, *sFRP1*, *sFRP5* and *DKK* was correlated with overall survival respectively in the univariate analysis. The silencing of other antagonists (such as sFRPs, Cerberus, Dab2 and Dkk ) could affect prognosis on patients with NSCLC concomitantly by the hypermethylation of *WIF1*. A further multivariate analysis involving *Wif-1*, *sFRPs*, *Cerberus*, *Dab2* and *Dkk* are required to find out whether Wif-1 methylation is independently or concomitantly associated with the prognosis in patients with NSCLC.

The results should be interpreted in view of certain limitations. First, only four studies were included in the meta-analysis, heterogeneity existed in some analysis which we used random effect model instead of fix effect model. Second, the included studies were retrospective and not randomized, however, most of them were of sufficiently quality (Newcastle-Ottawa Scale ≥ 7). Third, only studies in English and Chinese were included in the meta-analysis, eligible studies in other languages could be excluded. Fourth, smoker group was described as current and former smoker in one included study, [20] the finding could be confounded by former smoker. The result needs to be confirmed in more sensible way when more studies available.

In summary, the frequency of *WIF-1* hypermethylation significantly increased in NSCLC tumor compare with normal lung tissue. *WIF-1* gene is a potential marker for diagnosis of NSCLC and the prediction of prognosis in patients with NSCLC. *WIF-1* hypermethylation is predominant in squamous cell carcinoma (SCC), suggesting that *WIF-1* methylation contributes to the development of NSCLC, especially SCC.

## MATERIALS AND METHODS

We conducted a meta-analysis in accordance with the PRISMA guideline (Checklist S1).

### Search strategy

We performed a systematic electronic search in all available literature until July 2016 on PubMed, EMBASE, Web of Science and CNKI with no language limitations. We searched with the terms: “tumor or cancer or neoplasm or carcinoma” and “lung”, “methylation”, and “*WIF-1* or *Wnt inhibitory factor-1*”. Additional studies were identified through manually searching key journals and screening reference list of included studies. There were 11 articles identified from PubMed and one article from CNKI. A total of 12 articles were screened by article titles and abstracts.

### Selection criteria

Inclusion criteria were as follows: 1) Studies that investigated the relationship between *WIF-1* hypermethylation and the clinicopathological parameters of NSCLC; 2) *WIF-1* hypermethylation evaluated in the primary NSCLC tissues; 3) Studies provided information to estimate hazard ratio (HR) about 5-year overall survival (OS) and 95% confidence interval (CI). The studies were excluded based on the following criteria: 1) Reviews, case reports, letters, conference abstracts, editorials, expert opinion; 2) Studies in which same population or overlapping data were used.

### Data extraction and methodological assessment

We reviewed and extracted data from eligible studies. Any disagreements were resolved through discussion until a consensus was reached. The following items were collected from each study: the first author name, year of publication, number of cases, histology types of tumors, methylation detection method, methylation rate, 5-year overall survival rate. Data for study characteristics and clinical responses were summarized and organized into a table format.

For the methodological evaluation of the studies, we assessed and scored each publication independently according to the Newcastle Ottawa Quality Assessment Scale (NOQAS). The scale allocates a maximum of nine points for the quality of selection, comparability, exposure, and outcomes for study participants. The NOS scores ranged from 0 to 9, and a study with a score of 7 or more indicates a good quality.

### Statistical analysis

The meta-analysis was conducted using Review Manager 5.2 (Cochrane Collaboration, Software Update, Oxford, UK). Odds ratios (ORs) with its 95% confidence intervals were calculated. The *I^2^* statistics was used to examine the difference of study variability due to heterogeneity rather than chance, ranging from 0 to 100 percent. When the *I*^2^ value was below 50%, fixed effect model was used. When the *I*^2^ value was 50% or greater, a random effect model was used. The frequency of *WIF-1* hypermethylation was compared between normal lung tissue and NSCLC. In addition, we evaluated *WIF-1* methylation status in different histologic types of NSCLC as well as different smoking behavior. Two-sided *p* values less than 0.05 were considered statistically significant. Publication bias was assessed by using a method reported by Egger et al. [32]

## SUPPLEMENTARY MATERIALS CHECKLIST




