# Supplementary material for: Clinicopathological significance of WIF1 hypermethylation in NSCLC, a meta-analysis and literature review
Source: Oncotarget. 2016 Nov 29;8(2):2550–7. doi: 10.18632/oncotarget.13707 (PMC5356219; doi:10.18632/oncotarget.13707)
Supplement: Supplementary file 1 [file oncotarget-08-2550-s001.pdf]

Clinicopathological significance of *WIF1* hypermethylation in NSCLC, a meta-analysis and literature review

Supplementary Materials

Supplementary Checklist S1: PRISMA guideline checklist. See Supplementary_Checklist_S1
